# Efficient Production of Some Bioactive Depsides and Simple Phenolic Acids by Microshoots of *Aronia × Prunifolia* (Purple Aronia) Agitated Cultures as the Result of Feeding Strategy with Four Different Biogenetic Precursors

**DOI:** 10.3390/molecules29194622

**Published:** 2024-09-29

**Authors:** Paweł Kubica, Agnieszka Szopa, Adam Setkiewicz, Halina Ekiert

**Affiliations:** Department of Medicinal Plant and Mushroom Biotechnology, Jagiellonian University, Medical College, 30-688 Kraków, Poland; p.kubica@uj.edu.pl (P.K.); adam.setkiewicz@student.edu.pl (A.S.); halina.ekiert@uj.edu.pl (H.E.)

**Keywords:** in vitro cultures, purple aronia, phenylalanine, cinnamic acid, benzoic acid, caffeic acid, production of depsides, production of simple phenolic acids, HPLC analysis

## Abstract

A precursor feeding strategy was used for the first time in agitated microshoot cultures of *Aronia × prunifolia*. This strategy involved the addition of biogenetic precursors of simple phenolic acids (phenylalanine, cinnamic acid, and benzoic acid) and depsides (caffeic acid) into the culture media, with an assessment of its effect on the production of these bioactive compounds. The in vitro cultures were maintained in Murashige–Skoog medium (1 mg/L BAP and 1 mg/L NAA). Precursors at five concentrations (0.1, 0.5, 1.0, 5.0, and 10.0 mmol/L) were fed into the medium at the time of culture initiation (point “0”) and independently on the 10th day of growth cycles. The contents of 23 compounds were determined in methanolic extracts of biomass collected after 20 days of growth cycles using an HPLC method. All extracts contained the same four depsides (chlorogenic, neochlorogenic, rosmarinic, and cryptochlorogenic acids) and the same four simple phenolic acids (protocatechuic, vanillic, caffeic, and syringic acids). Chlorogenic and neochlorogenic acids were the predominant compounds in all extracts (max. 388.39 and 263.54 mg/100 g d.w.). The maximal total contents of all compounds were confirmed after feeding with cinnamic acid (5 mmol/L, point “0”) and caffeic acid (10 mmol/L, point “0”), which caused a 2.68-fold and 2.49-fold increase in the contents of the estimated compounds vs. control cultures (603.03 and 558.48 mg/100 g d.w., respectively). The obtained results documented the efficacy of the precursor feeding strategy in enhancing the production of bioactive compounds in agitated cultures of *A. × prunifolia* and suggest a potential practical application value.

## 1. Introduction

Phenolic acids, including depsides (compounds containing two or more molecules of phenolic acids linked by an ester bond), are a group of plant-derived polyphenol compounds known for their strong antioxidant properties [1]. These antioxidant properties are decisive for the valuable biological activity of this group of plant metabolites and their usefulness in the prevention and treatment of various diseases of civilization, such as stroke, myocardial infarction, metabolic diseases (e.g., obesity, diabetes, atherosclerosis), and common neurodegenerative diseases [1,2,3,4,5,6,7].

Apart from these invaluable activities, phenolic acids (including depsides) show numerous other well-documented biological activities. They have been proven to possess anti-inflammatory, immunostimulating, hepatoprotective, as well as anticoagulant and cytotoxic properties [2,5,8].

Plant antioxidants, including phenolic acids, are of particular interest not only to the pharmaceutical and healthy food industries but also to the cosmetic industry. In cosmetology, the antiaging properties of these compounds are of the greatest importance [3]. Pharmaceutical, food, and cosmetic companies are continuously searching for new natural sources of antioxidants.

Many investigations have documented that certain groups of antioxidants can be produced in high amounts in in vitro cultures of various plant species [9]. Our team’s research has also demonstrated the high biosynthetic potential of plant cells cultured in vitro to produce various groups of antioxidants with polyphenolic structures, such as phenolic acids [10]. Currently, some cosmetic products and food supplements are produced on a commercial scale in European countries, the USA, and Korea using plant in vitro technology [11,12,13].

The aim of the present study was to propose *Aronia × prunifolia* in vitro cultures as a potential new, rich source of antioxidants—simple phenolic acids and depsides. We expected that, by applying the well-known feeding strategy with biogenetic precursors used in plant biotechnology, the obtained results would have potential practical application value.

*A. × prunifolia* (Marsh.) Rehd. (purple aronia) is a natural North American hybrid of two species of the genus *Aronia*: *A. melanocarpa* (Michx.) Elliot (black aronia) and *A. arbutifolia* (L.) Pers. (red aronia), also native to North America. This hybrid is cultivated in some European countries, including Poland, but mostly as an ornamental plant. One publication has documented the spontaneous occurrence and dispersion of this hybrid in Poland [14].

Phytochemical studies conducted by our team on both the fruits and leaves of *Aronia* plants growing in the Arboretum of the Warsaw University of Life Sciences in Rogów (Poland) revealed high levels of various polyphenolic antioxidants: phenolic acids, anthocyanins, and flavonoids in the tested plant material [15].

The studies documented that fruit extracts of *A. × prunifolia* contained three cyanidin glycosides (galactoside, arabinoside, and glucoside), with a total content amounting to 470 mg/100 g dry weight (d.w.). The flavonoid group was represented solely by quercetin (44.3 mg/100 g d.w.), while among phenolic acids, chlorogenic acid (273.5 mg/100 g d.w.) and neochlorogenic acid (212.6 mg/100 g d.w.) were predominant in the fruit extracts. These were accompanied by smaller amounts of rosmarinic acid, protocatechuic acid, and 3,4-dihydroxyphenylacetic acid (9.2, 4.4, and 4.3 mg/100 g d.w., respectively). The total content of these compounds in fruit extracts was lower compared to leaf extracts (503.9 vs. 1175.8 mg/100 g d.w.). The flavonoid content was also lower in fruit extracts (44.3 mg/100 g d.w.) compared to leaf extracts (786.4 mg/100 g d.w.). On the other hand, among anthocyanins in leaf extracts, only cyanidin galactoside was identified (1.2 mg/100 g d.w.) [15].

The chemical composition of fruits from purple aronia, sourced from locations in the United States, has also been investigated by the Department of Natural Sciences, University of Connecticut (USA). The studies documented the presence of several depsides—chlorogenic and neochlorogenic acids—as well as flavonoids, anthocyanins, and proanthocyanidins in the fruit extracts. The main compounds identified were the above-mentioned depsides and quercetin glycosides [16]. However, detailed professional phytochemical studies of this plant are, however, still awaited.

The in vitro cultures of *A. × prunifolia* were established by our team in 2013. Since then, our investigations have concentrated on the biosynthetic potential of callus and microshoot cultures of *A. × prunifolia* (including agar stationary callus and microshoot cultures, as well as agitated cultures of microshoots). The results have documented a high capacity of these in vitro-cultured cells to produce phenolic acids, including depsides [17,18,19].

In detail, we examined the effect of several concentrations of the chosen regulators of plant growth and development: BAP (6-benzylaminopurine) and NAA (1-naphthaleneacetic acid) in Murashige–Skoog (MS) medium [20] on the production of phenolic acids. This investigation was conducted both in stationary (callus and microshoot) cultures [18] and in agitated microshoot cultures of *A. × prunifolia* [17] and also assessed the impact of monochromatic fluorescent light on the production of these compounds in stationary agar cultures of microshoots [19].

Apart from the in vitro culture conditions tested so far by our team, many other chemical, physical, and genetic factors known in plant biotechnology influence the production of secondary metabolites in in vitro cultures [21]. Common and effective methods include supplementing culture media with biogenetic precursors of bioactive metabolites [21] abiotic and biotic elicitors, and genetic transformation with *Rhizobium rhizogenes* (earlier *Agrobacterium rhizogenes*) [21,22]

Encouraged by the promising results obtained by our team using the precursor feeding strategy—entailing the addition of biogenetic precursors of phenolic acids (phenylalanine, cinnamic acid, and benzoic acid) and depsides (caffeic acid) to culture media in agitated cultures of *A. melanocarpa* and *A. arbutifolia*—we decided to conduct similar experiments on agitated microshoot cultures of *A. × prunifolia* [23].

Our decision was also supported by the attractive results currently obtained by our team using precursor feeding in vitro cultures of other plant species (*Ginkgo biloba*, *Ruta graveolens, Hypericum perforatum cvs*, and *Scutellaria lateriflora*) [24,25,26,27].

In currently performed by our team biotechnological investigations, *A. × prunifolia* cultures were grown in an MS medium variant (containing 1 mg/L BAP and 1 mg/L NAA), selected by our team based on earlier studies with aronia plant in vitro cultures as a suitable “universal” production medium for microshoot cultures of black, red, and purple aronias. This MS medium variant was supplemented with three biogenetic precursors of simple phenolic acids—phenylalanine, cinnamic acid, and benzoic acid—and caffeic acid (as a depside precursor) at two time points (initial culture and on the 10th day of culture). Each precursor was tested at five concentrations (0.1, 0.5, 1.0, 5.0, and 10.0 mmol/L). The results of these extensive experimental studies are presented below.

## 2. Results

### 2.1. Unfed Control Cultures

**Figure 1:**
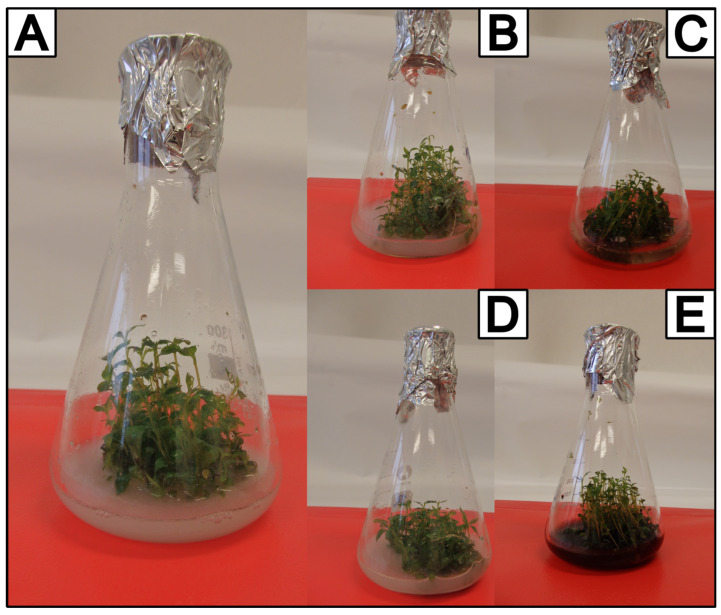
In vitro cultures of *Aronia × prunifolia* after a 20-day growth cycle. (**A**) Control culture and experimental cultures after the addition of (**B**) 5 mmol/L phenylalanine; (**C**) 5 mmol/L cinnamic acid; (**D**) 5 mmol/L benzoic acid; and (**E**) 5 mmol/L caffeic acid.

The 20-day growth of *A. × prunifolia* shoot cultures resulted in a 6.34-fold increase in dry biomass (Table 1, Figure 1). Analysis of methanolic extracts from the biomass revealed the presence of a total of eight phenolic acids: four depsides (chlorogenic, neochlorogenic, rosmarinic, and cryptochlorogenic acids) and four simple phenolic acids (protocatechuic, vanillic, caffeic, and syringic acids). The remaining 15 tested phenolic acids were not identified.

Of these, chlorogenic acid and neochlorogenic acid were accumulated in the greatest amounts (105.30 and 90.01 mg/100 g d.w., respectively). The contents of the other compounds varied, ranging from 0.83 mg/100 g d.w. (vanillic acid) to 11.57 mg/100 g d.w. (syringic acid). The remaining two depsides accumulated at similar levels (rosmarinic acid: 6.29 mg/100 g d.w., and cryptochlorogenic acid: 7.01 mg/100 g d.w.). The total content of all eight compounds was 224.74 mg/100 g d.w.

**Table 1:** Dry biomass increments ± SD of *A. × prunifolia* microshoots cultured in vitro on media supplemented with precursors.

Precursor Added [mmol/L]	Dry Biomass Increments		Precursor Added [mmol/L]	Dry Biomass Increments	
Phenylalanine	Point “0”	10th Day	Benzoic Acid	Point “0”	10th Day
0.1	7.54 ± 1.55	8.55 ± 0.84 *	0.1	6.33 ± 0.72	6.21 ± 1.08
0.5	7.44 ± 0.48	8.87 ± 1.04 *	0.5	7.65 ± 0.45 *	7.64 ± 0.72 *
1	8.54 ± 1.03 *	9.01 ± 1.07 *	1	6.33 ± 1.01	6.21 ± 0.78
5	6.32 ± 0.78	7.56 ± 0.98	5	2.33 ± 0.77 *	4.59 ± 0.81 *
10	2.87 ± 0.88 *	5.56 ± 0.77	10	1.96 ± 0.44 *	5.17 ± 1.31 *
**Cinnamic Acid**	**Point “0”**	**10th day**	**Caffeic Acid**	**Point “0”**	**10th Day**
0.1	8.65 ± 0.55 *	8.22 ± 1.07 *	0.1	8.45 ± 1.77 *	7.87 ± 0.93 *
0.5	8.02 ± 0.85 *	8.14 ± 1.11 *	0.5	7.88 ± 1.07 *	7.06 ± 0.73 *
1	7.52 ± 0.77 *	7.87 ± 1.65 *	1	7.21 ± 1.38 *	6.61 ± 0.72
5	2.01 ± 0.37 *	5.66 ± 0.74 *	5	6.45 ± 0.76	5.77 ± 0.94
10	1.54 ± 0.87 *	4.01 ± 1.08 *	10	2.02 ± 0.87 *	5.37 ± 1.02 *

Control cultures—dry biomass increments: 6.34 ± 1.45. * *p* < 0.05.

### 2.2. Precursor-Fed Experimental Cultures

Cultures fed with biogenetic precursors, especially when added at the initial point (“0”) and at the highest concentration (10 mmol/L), showed inhibition of biomass growth after the 20-day growth cycle. In cinnamic acid-fed and benzoic acid-fed cultures, this effect was observed at a precursor concentration of 5 mmol/L (Table 1).

The analyzed extracts contained the same composition of phenolic acids (both depsides and simple phenolic acids) as the unfed control cultures. However, differences were noted in the quantities of particular compounds and their total content compared to the control cultures.

Two depsides, chlorogenic acid and neochlorogenic acid, predominated in all extracts from the experimental cultures, just as they did in the control cultures (Table 2, Table 3, Table 4, Table 5, Table 6, Table 7, Table 8 and Table 9).

#### 2.2.1. Phenylalanine-Fed Cultures

##### Biomass Increments

Shoot biomass increments in cultures fed with phenylalanine at concentrations of 0.1 to 1 mmol/L ranged from 7.44-fold to 9.01-fold and were higher than in the control cultures. Biomass increments were also greater compared to control cultures (7.56-fold) when this precursor was added at a higher concentration of 5 mmol/L on the 10th day of culture. Only the highest concentration of phenylalanine (10 mmol/L) inhibited biomass growth, resulting in a 2.87-fold increase at the initial point (“0”) and a 5.56-fold increase on the 10th day (Table 1).

### 2.3. Accumulation of Phenolic Acids

#### 2.3.1. Point “0”: Culture Initiation

Two depsides, chlorogenic acid and neochlorogenic acid, predominated quantitatively in the analyzed extracts. Their contents varied depending on the phenylalanine concentration, changing 3.15-fold and 3.0-fold, respectively. Chlorogenic acid ranged from 88.43 mg/100 g d.w. (10 mmol/L) to 278.96 mg/100 g d.w. (5 mmol/L), while neochlorogenic acid ranged from 40.86 mg/100 g d.w. (10 mmol/L) to 122.49 mg/100 g d.w. (1 mmol/L). The other two depsides, rosmarinic acid and cryptochlorogenic acid, were accumulated in much smaller amounts, with rosmarinic acid reaching a maximum of 22.24 mg/100 g d.w. and cryptochlorogenic acid reaching a maximum of 12.92 mg/100 g d.w. The contents of the remaining compounds, i.e., simple phenolic acids, did not exceed 18 mg/100 g d.w.: syringic acid (maximum 17.80 mg/100 g d.w.), caffeic acid (maximum 12.32 mg/100 g d.w.), protocatechuic acid (maximum 7.53 mg/100 g d.w.), and vanillic acid (maximum 4.13 mg/100 g d.w.) (Table 2).

**Figure 2:**
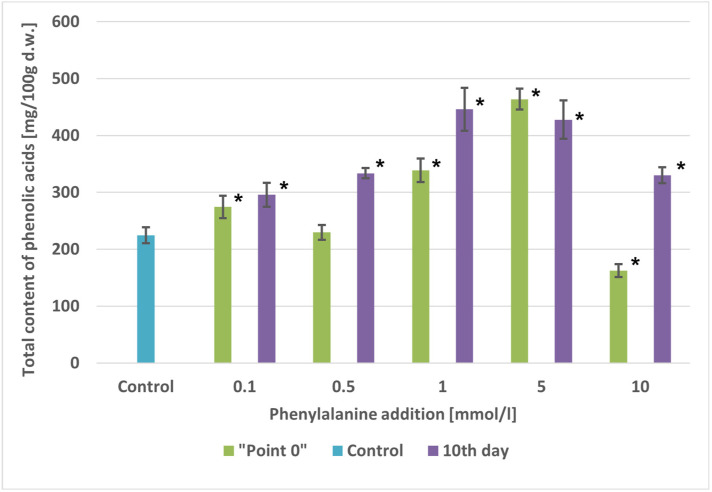
Comparison of total phenolic acids content [mg/100 g d.w.] in extracts of *A. × prunifolia* in vitro cultures supplemented with phenylalanine. * *p* < 0.05.

The total contents of phenolic acids were 1.02-fold to 2.06-fold higher than in the control cultures (Figure 2). These contents ranged from 229.74 mg/100 g d.w. (0.5 mmol/L) to 463.77 mg/100 g d.w. (5 mmol/L), depending on the precursor concentration. At the maximum tested concentration of phenylalanine (10 mmol/L), there was a 1.4-fold decrease in the total content of compounds compared to the control cultures. This decrease was attributed to significant inhibition of biomass growth (Figure 2).

**Table 2:** Content of phenolic acids [mg/100 g d.w.] ± SD in extracts of *A. × prunifolia* in vitro cultures supplemented with phenylalanine at “point 0” (establishment of agitated culture). * *p* < 0.05.

Estimated Compounds	Control	Phenylalanine Concentration [mmol/L] Added at “Point 0”				
		0.1	0.5	1	5	10
**Neochlorogenic acid**	90.01 ± 8.96	99.16 ± 1.30	81.93 ± 7.47	122.49 ± 4.25 *	114.35 ± 2.43 *	40.86 ± 4.44 *
**Protocatechuic acid**	1.14 ± 0.08	2.61 ± 0.28 *	6.36 ± 0.22 *	3.13 ± 0.26 *	7.53 ± 0.69 *	4.04 ± 0.47 *
**Chlorogenic acid**	105.30 ± 11.17	133.36 ± 15.64 *	99.44 ± 3.79	161.26 ± 12.56 *	278.96 ± 13.28 *	88.43 ± 4.43
**Vanillic acid**	0.83 ± 0.05	1.45 ± 0.09 *	1.44 ± 0.02 *	1.60 ± 0.11 *	4.13 ± 0.04 *	3.32 ± 0.35 *
**Caffeic acid**	2.58 ± 0.25	5.40 ± 0.23 *	6.70 ± 0.49 *	6.52 ± 0.75 *	12.32 ± 0.56 *	2.58 ± 0.26
**Syringic acid**	11.57 ± 0.20	14.37 ± 1.03 *	1.29 ± 0.06 *	12.57 ± 1.49	17.80 ± 0.58 *	4.99 ± 0.32 *
**Rosmarinic acid**	6.29 ± 0.59	12.33 ± 1.11 *	19.65 ± 0.77 *	22.24 ± 0.91 *	19.65 ± 0.27 *	9.25 ± 0.83
**Cryptochlorogenic acid**	7.01 ± 0.69	5.62 ± 0.32	12.92 ± 0.24 *	9.04 ± 0.33 *	9.04 ± 0.49 *	8.92 ± 0.21 *

#### 2.3.2. Tenth Day after Culture Initiation

The main compounds in the tested extracts were identified as chlorogenic acid and neochlorogenic acid, the same depsides that predominated in extracts from the control cultures, showing a 1.84-fold and 2.26-fold increase, respectively. Depending on the phenylalanine concentration, their contents ranged from 124.88 mg/100 g d.w. (0.1 mmol/L) to 230.08 mg/100 g d.w. (5 mmol/L) for chlorogenic acid and from 64.80 mg/100 g d.w. (10 mmol/L) to 146.65 mg/100 g d.w. (1 mmol/L) for neochlorogenic acid (Table 3).

The remaining depsides accumulated in similar quantities but on a different scale: rosmarinic acid reached a maximum of 41.27 mg/100 g d.w., and cryptochlorogenic acid reached a maximum of 36.26 mg/100 g d.w. The contents of three simple phenolic acids were below 12 mg/100 g d.w., with specific figures as follows: protocatechuic acid—4.66 mg/100 g d.w., vanillic acid—5.00 mg/100 g d.w., and caffeic acid—11.20 mg/100 g d.w. Only syringic acid had a higher content, reaching a maximum of 28.43 mg/100 g d.w.

The addition of phenylalanine to the culture medium increased the total contents of phenolic acids by 1.31-fold to 1.98-fold compared to the control culture, depending on the precursor concentration. The smallest increase in phenolic acid accumulation was observed at a precursor concentration of 0.1 mmol/L (295.65 mg/100 g d.w.), while the largest increase was at 1 mmol/L (445.99 mg/100 g d.w.) (Figure 2).

**Table 3:** Content of phenolic acids [mg/100 g d.w.] ± SD in extracts of *A. × prunifolia* in vitro cultures supplemented with phenylalanine on the 10th day of the growth cycle. * *p* < 0.05.

Estimated Compounds	Control	Phenylalanine Concentration [mmol/L] Added at 10th Day				
		0.1	0.5	1	5	10
**Neochlorogenic acid**	90.01 ± 8.96	104.28 ± 12.06	117.49 ± 2.64 *	146.65 ± 7.9 *	111.98 ± 13.08 *	64.80 ± 1.22 *
**Protocatechuic acid**	1.14 ± 0.08	2.87 ± 0.05 *	2.97 ± 0.29 *	3.84 ± 0.42 *	4.66 ± 0.16 *	3.61 ± 0.31 *
**Chlorogenic acid**	105.30 ± 11.17	124.88 ± 7.12 *	132.65 ± 1.84 *	186.12 ± 21.21 *	230.08 ± 14.73 *	220.43 ± 8.53 *
**Vanillic acid**	0.83 ± 0.05	1.79 ± 0.03 *	2.30 ± 0.11 *	3.58 ± 0.29 *	3.24 ± 0.10 *	5.00 ± 0.27 *
**Caffeic acid**	2.58 ± 0.25	8.38 ± 0.54 *	8.51 ± 0.45 *	11.20 ± 0.12 *	7.37 ± 0.35 *	5.22 ± 0.61 *
**Syringic acid**	11.57 ± 0.20	19.83 ± 0.46 *	17.69 ± 1.73 *	28.43 ± 1.52 *	16.56 ± 1.64 *	8.41 ± 0.70
**Rosmarinic acid**	6.29 ± 0.59	23.42 ± 0.35 *	25.87 ± 1.21 *	29.91 ± 3.36 *	41.27 ± 3.32 *	12.49 ± 1.35 *
**Cryptochlorogenic acid**	7.01 ± 0.69	10.20 ± 0.27 *	26.10 ± 0.91 *	36.26 ± 3.17 *	12.56 ± 0.44 *	10.22 ± 1.11

#### 2.3.3. Cinnamic Acid-Fed Cultures

##### Biomass Increments

Feeding cinnamic acid at lower concentrations (0.1–1 mmol/L) promoted shoot biomass growth, with increments greater than those in the control cultures (7.52–8.65-fold). High concentrations of this precursor (5 and 10 mmol/L) added at point “0” caused significant inhibition of biomass growth, resulting in only a 2.01-fold and 1.54-fold increase, respectively. When this precursor was added to the media on the 10th day, biomass increments were also low: 5.66-fold and 4.01-fold, respectively (Table 1).

### 2.4. Accumulation of Phenolic Acids

#### 2.4.1. Point “0”: Culture Initiation

Depsides, specifically chlorogenic acid and neochlorogenic acid, were the main compounds in the studied extracts. The content of chlorogenic acid varied widely, more than 11-fold, depending on the precursor concentration, ranging from 34.61 mg/100 g d.w. (10 mmol/L) to 388.39 mg/100 g d.w. (5 mmol/L). On the other hand, neochlorogenic acid levels increased approximately 3-fold (2.96-fold), from 49.41 mg/100 g d.w. (10 mmol/L) to 146.52 mg/100 g d.w. (1 mmol/L) (Table 4).

Rosmarinic acid amounts fluctuated widely, from 0.53 to 42.41 mg/100 g d.w., while cryptochlorogenic acid levels ranged from 6.61 to 14.32 mg/100 g d.w.

The maximum contents of the two remaining phenolic acids were 13.99 mg/100 g d.w. for protocatechuic acid and 7.14 mg/100 g d.w. for vanillic acid. The maximum contents of the two compounds were nearly identical: caffeic acid at 19.87 mg/100 g d.w. and syringic acid at 20.45 mg/100 g d.w.

**Figure 3:**
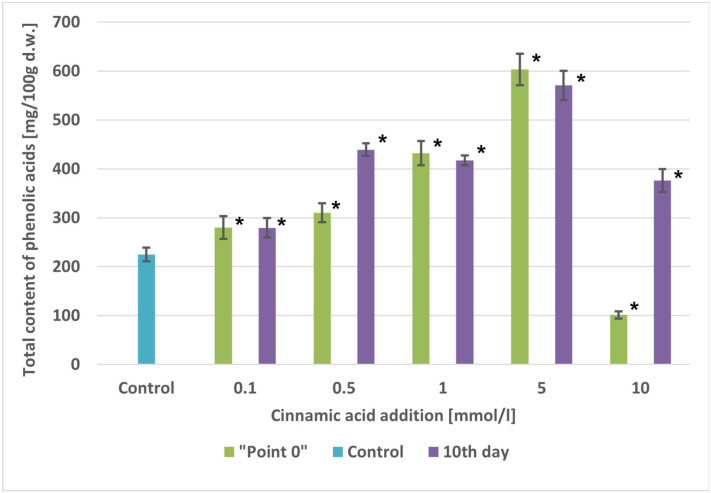
Comparison of total phenolic acid content [mg/100 g d.w.] in extracts of *A. × prunifolia* in vitro cultures supplemented with cinnamic acid. * *p* < 0.05.

The total contents of phenolic acids were documented to be 1.24-fold to 2.68-fold higher than in the control cultures, ranging from 279.73 mg/100 g d.w. (0.1 mmol/L) to 603.03 mg/100 g d.w. (5 mmol/L), depending on cinnamic acid concentration. Only at the highest tested concentration of cinnamic acid (10 mmol/L) was there a 2.20-fold decrease in the total content of the tested compounds, which was attributed to the suppressive effect of cinnamic acid at this concentration on biomass increments (Table 1, Figure 3).

**Table 4:** Content of phenolic acids [mg/100 g d.w.] ± SD in extracts of *A. × prunifolia* in vitro cultures supplemented with cinnamic acid at “point 0”. * *p* < 0.05.

Estimated Compounds	Control	Cinnamic Acid Concentration [mmol/L] Added at “Point 0”				
		0.1	0.5	1	5	10
**Neochlorogenic acid**	90.01 ± 5.41	118.99 ± 9.38 *	111.21 ± 12.97 *	146.52 ± 10.54 *	100.78 ± 7.48 *	49.41 ± 1.88 *
**Protocatechuic acid**	1.14 ± 0.04	2.48 ± 0.21 *	3.12 ± 0.21 *	3.59 ± 0.12 *	13.99 ± 0.43 *	3.55 ± 0.22 *
**Chlorogenic acid**	105.30 ± 10.66	105.20 ± 11.16	156.60 ± 4.98 *	222.79 ± 9.43 *	388.39 ± 16.83 *	34.61 ± 4.04 *
**Vanillic acid**	0.83 ± 0.06	2.02 ± 0.15 *	2.13 ± 0.21 *	2.05 ± 0.13 *	7.14 ± 0.25 *	2.65 ± 0.22 *
**Caffeic acid**	2.58 ± 0.12	6.09 ± 0.73 *	7.91 ± 0.09 *	8.48 ± 0.68 *	19.87 ± 1.35 *	5.76 ± 0.43 *
**Syringic acid**	11.57 ± 0.93	15.84 ± 0.40	20.45 ± 0.71 *	14.01 ± 1.40	16.14 ± 1.29 *	1.57 ± 0.10 *
**Rosmarinic acid**	6.29 ± 0.65	18.68 ± 0.53 *	0.53 ± 0.03 *	28.16 ± 2.51 *	42.41 ± 3.04 *	1.23 ± 0.11 *
**Cryptochlorogenic acid**	7.01 ± 0.21	10.43 ± 0.58 *	8.52 ± 0.23	6.61 ± 0.11	14.32 ± 1.32 *	2.36 ± 0.11 *

#### 2.4.2. Tenth Day after Culture Initiation

Of all the phenolic acids tested in methanolic biomass extracts, two compounds, chlorogenic acid and neochlorogenic acid, were accumulated in the greatest amounts. These are the same depsides that predominated in the shoots from the experimental cultures (point “0”). Their amounts changed similarly, ranging from 2.12-fold to 2.37-fold depending on precursor concentration, from 142.46 mg/100 g d.w. (0.1 mmol/L) to 337.59 mg/100 g d.w. (5 mmol/L) for chlorogenic acid, and from 66.34 mg/100 g d.w. (10 mmol/L) to 140.79 mg/100 g d.w. (5 mmol/L) for neochlorogenic acid (Table 5).

The amounts of rosmarinic acid accumulated were of a much different order of magnitude, reaching a maximum of 31.64 mg/100 g d.w.). The accumulation of the next depside, cryptochlorogenic acid, was even lower, approximately 2-fold, at 16.23 mg/100 g d.w. Simple phenolic acids reached the following maximum contents: syringic acid at 27.75 mg/100 g d.w., caffeic acid at 11.92 mg/100 g d.w., protocatechuic acid at 4.85 mg/100 g d.w., and vanillic acid at 4.28 mg/100 g d.w.

After the addition of cinnamic acid, the total contents of the accumulated phenolic acids increased from 1.24-fold to 2.54-fold compared with the control cultures, ranging from 279.35 mg/100 g d.w. (0.1 mmol/L) to 570.57 mg/100 g d.w. (5 mmol/L) (Figure 3).

**Table 5:** Content of phenolic acids [mg/100 g d.w.] ± SD in extracts of *A. × prunifolia* in vitro cultures supplemented with cinnamic acid on the 10th day of the growth cycle. * *p* < 0.05.

Estimated Compounds	Control	Cinnamic Acid Concentration [mmol/L] Added at 10th Day				
		0.1	0.5	1	5	10
**Neochlorogenic acid**	90.01 ± 5.41	86.67 ± 9.27	135.63 ± 2.23 *	91.76 ± 1.73	140.79 ± 12.04 *	66.34 ± 2.02 *
**Protocatechuic acid**	1.14 ± 0.04	2.72 ± 0.09 *	3.52 ± 0.23 *	3.28 ± 0.08 *	4.85 ± 0.24 *	4.57 ± 0.53 *
**Chlorogenic acid**	105.30 ± 10.66	142.46 ± 6.85 *	228.36 ± 4.07 *	265.10 ± 4.85 *	337.59 ± 12.11 *	252.49 ± 17.47 *
**Vanillic acid**	0.83 ± 0.06	2.88 ± 0.12 *	1.52 ± 0.13 *	2.56 ± 0.16 *	2.34 ± 0.09 *	4.28 ± 0.42 *
**Caffeic acid**	2.58 ± 0.12	6.66 ± 0.73 *	6.60 ± 0.26 *	7.10 ± 0.53 *	11.92 ± 0.47 *	7.52 ± 0.47 *
**Syringic acid**	11.57 ± 0.93	20.53 ± 2.09 *	20.23 ± 2.38 *	21.13 ± 1.27 *	27.75 ± 0.96 *	5.50 ± 0.18 *
**Rosmarinic acid**	6.29 ± 0.65	10.24 ± 0.18 *	27.07 ± 3.22 *	17.89 ± 0.56 *	31.64 ± 2.68 *	30.92 ± 2.01 *
**Cryptochlorogenic acid**	7.01 ± 0.21	7.17 ± 0.31	16.23 ± 0.20 *	8.52 ± 0.63 *	13.69 ± 1.05 *	4.20 ± 0.21 *

#### 2.4.3. Benzoic Acid-Fed Cultures

##### Biomass Increments

Shoot biomass increments in the experimental cultures with the addition of benzoic acid at concentrations of 0.1 and 1 mmol/L were comparable to those in the control cultures. However, this precursor at 0.5 mmol/L caused a 7.65-fold stimulation of biomass growth at point “0” and a 7.64-fold increase by the 10th day.

The highest tested concentrations of benzoic acid (5 and 10 mmol/L) induced a significant suppression of biomass growth, which was particularly noticeable after precursor addition at point “0” (resulting in only a 2.33-fold and 1.96-fold increase) (Table 1).

### 2.5. Accumulation of Phenolic Acids

#### 2.5.1. Point “0”: Culture Initiation

**Figure 4:**
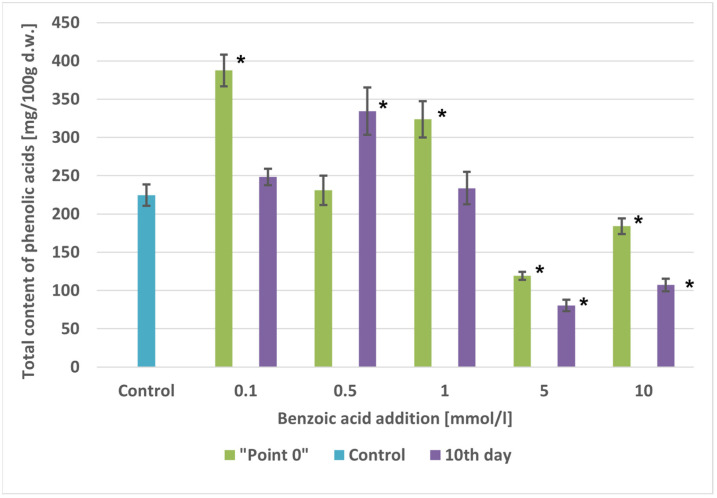
Comparison of total phenolic acids content [mg/100 g d.w.] in extracts of *A. × prunifolia* in vitro cultures supplemented with benzoic acid. * *p* < 0.05.

The highest tested concentrations of benzoic acid (5 and 10 mmol/L) caused a decrease in the contents of several phenolic acids and, consequently, a reduction in the total content by 1.89-fold (5 mmol/L) and 1.22-fold (10 mmol/L), respectively, compared with the control cultures. This decrease was due to a drastic reduction in biomass growth (Table 1). At lower concentrations of benzoic acid (0.1–1.0 mmol/L), there was a slight increase in the total content of phenolic acids, ranging from 1.03-fold (0.5 mmol/L) to 1.72-fold (0.1 mmol/L), with amounts of 230.78 and 387.49 mg/100 g d.w., respectively (Table 6 and Figure 4).

Two depsides, chlorogenic acid and neochlorogenic acid, were the main accumulated compounds. Their contents varied similarly, increasing 4.62-fold and 4.73-fold depending on benzoic acid concentration, ranging from 36.29 mg/100 g d.w. (5 mmol/L) to 167.66 mg/100 g d.w. (0.1 mmol/L) and from 31.37 mg/100 g d.w. (5 mmol/L) to 148.49 mg/100 g d.w. (0.1 mmol/L), respectively. The maximum contents of rosmarinic acid (31.68 mg/100 g d.w.) and cryptochlorogenic acid (11.01 mg/100 g d.w.) were considerably lower.

Among simple phenolic acids, syringic acid predominated (maximum 22.80 mg/100 g d.w.), while the contents of caffeic, protocatechuic, and vanillic acids ranged from approximately 1 mg/100 g d.w. to approximately 10 mg/100 g d.w. (Table 6).

**Table 6:** Content of phenolic acids [mg/100 g d.w.] ± SD in extracts of *A. × prunifolia* in vitro cultures supplemented with benzoic acid at “point 0”. * *p* < 0.05.

Estimated Compounds	Control	Benzoic Acid Concentration [mmol/L] Added at “Point 0”				
		0.1	0.5	1	5	10
**Neochlorogenic acid**	90.01 ± 2.77	148.49 ± 6.76 *	84.75 ± 9.75	115.99 ± 12.59 *	31.37 ± 2.68 *	47.08 ± 4.47 *
**Protocatechuic acid**	1.14 ± 0.07	2.27 ± 0.06 *	2.31 ± 0.14	3.01 ± 0.03 *	2.57 ± 0.07 *	3.51 ± 0.41 *
**Chlorogenic acid**	105.30 ± 4.9	167.66 ± 9.82 *	102.41 ± 6.92	138.29 ± 5.85 *	36.29 ± 0.78 *	93.52 ± 2.25
**Vanillic acid**	0.83 ± 0.07	0.98 ± 0.07	1.30 ± 0.02 *	2.47 ± 0.17 *	2.16 ± 0.22 *	2.54 ± 0.20 *
**Caffeic acid**	2.58 ± 0.11	6.37 ± 0.49 *	5.92 ± 0.16 *	9.87 ± 0.63 *	1.40 ± 0.13	7.44 ± 0.68 *
**Syringic acid**	11.57 ± 0.66	22.80 ± 2.09 *	10.96 ± 0.56 *	19.47 ± 1.26 *	3.26 ± 0.11	7.25 ± 0.46 *
**Rosmarinic acid**	6.29 ± 0.61	29.89 ± 1.09 *	12.36 ± 1.33 *	23.49 ± 1.69 *	31.68 ± 0.44 *	17.50 ± 1.22 *
**Cryptochlorogenic acid**	7.01 ± 0.33	9.03 ± 0.29	10.77 ± 0.22 *	11.01 ± 1.30 *	10.43 ± 0.75 *	5.23 ± 0.52

#### 2.5.2. Tenth Day after Culture Initiation

In cultures fed with benzoic acid on the 10th day of growth, the highest precursor concentrations (5 and 10 mmol/L) significantly reduced the contents of individual phenolic acids and their total content by 2.79-fold (5 mmol/L) and 2.09-fold (10 mmol/L), respectively. The highest tested concentrations of benzoic acid (5 and 10 mmol/L) also suppressed microshoot biomass growth (Table 1 and Table 7 and Figure 4).

Feeding the precursor at the other tested concentrations caused a slight increase in the total content of phenolic acids, ranging from 1.10-fold (0.1 mmol/L) to 1.49-fold (0.5 mmol/L), with values reaching 248.28 and 323.61 mg/100 g d.w., respectively.

The contents of the predominant compounds, chlorogenic acid and neochlorogenic acid, increased within a wide range, 11.92-fold and 7.58-fold, respectively, ranging from 11.59 mg/100 g d.w. (10 mmol/L) to 138.12 mg/100 g d.w. (0.5 mmol/L) and from 17.31 mg/100 g d.w. (10 mmol/L) to 131.16 mg/100 g d.w. (0.5 mmol/L). The maximum accumulated contents of the remaining depsides, rosmarinic acid and cryptochlorogenic acid, were 23.24 and 8.94 mg/100 g d.w., respectively.

Among simple phenolic acids, syringic acid was accumulated in the greatest amounts (maximum 20.58 mg/100 g d.w.). The contents of the other three compounds—caffeic, vanillic, and protocatechuic acids—did not exceed 7.50 mg/100 g d.w.

**Table 7:** Content of phenolic acids [mg/100 g d.w.] ± SD in extracts of *A. × prunifolia* in vitro cultures supplemented with benzoic acid on the 10th day of the growth cycle. * *p* < 0.05.

Estimated Compounds	Control	Benzoic Acid Concentration [mmol/L] Added at 10th Day				
		0.1	0.5	1	5	10
**Neochlorogenic acid**	90.01 ± 2.77	88.86 ± 4.98	131.16 ± 12.95 *	96.86 ± 11.06	41.72 ± 4.97 *	17.31 ± 1.10 *
**Protocatechuic acid**	1.14 ± 0.07	2.47 ± 0.14 *	2.71 ± 0.20 *	2.72 ± 0.19 *	1.60 ± 0.15	2.57 ± 0.21 *
**Chlorogenic acid**	105.30 ± 4.9	123.80 ± 4.18 *	138.12 ± 14.67 *	87.39 ± 8.14	11.59 ± 1.17 *	55.25 ± 5.56 *
**Vanillic acid**	0.83 ± 0.07	1.64 ± 0.07 *	2.63 ± 0.25 *	1.96 ± 0.21 *	0.53 ± 0.01	1.79 ± 0.05 *
**Caffeic acid**	2.58 ± 0.11	5.56 ± 0.22 *	7.20 ± 0.31 *	7.11 ± 0.61 *	3.21 ± 0.33	2.52 ± 0.04
**Syringic acid**	11.57 ± 0.66	5.15 ± 0.11 *	20.58 ± 0.40 *	14.00 ± 0.48 *	4.96 ± 0.10 *	5.82 ± 0.41 *
**Rosmarinic acid**	6.29 ± 0.61	13.75 ± 0.38 *	23.24 ± 1.42 *	14.72 ± 0.19 *	9.65 ± 0.69	15.90 ± 0.37 *
**Cryptochlorogenic acid**	7.01 ± 0.33	7.06 ± 0.51	8.67 ± 0.75 *	8.94 ± 0.19 *	7.17 ± 0.09	6.21 ± 0.50

#### 2.5.3. Caffeic Acid-Fed Cultures

##### Biomass Increments

Feeding caffeic acid to culture media at concentrations ranging from 0.1 to 1.0 mmol/L produced a beneficial effect on dry biomass growth, resulting in increases of 6.61-fold to 8.45-fold. The highest tested concentration of this precursor (10 mmol/L) suppressed biomass growth, with only a 2.02-fold increase observed after precursor addition to the medium at point ”0” (Table 1).

### 2.6. Accumulation of Phenolic Acids

#### 2.6.1. Point “0”: Culture Initiation

Chlorogenic acid and neochlorogenic acid were the main compounds accumulated in biomass extracts from cultures maintained with the addition of caffeic acid as a depside precursor. The contents of these compounds increased 1.46-fold and 2.19-fold, respectively, depending on caffeic acid concentration, ranging from 123.45 mg/100 g d.w. (0.1 mmol/L) to 180.22 mg/100 g d.w. (10 mmol/L) for chlorogenic acid and from 120.24 mg/100 g d.w. (0.1 mmol/L) to 263.54 mg/100 g d.w. (10 mmol/L) for neochlorogenic acid (Table 8).

Rosmarinic acid contents ranged from 20.53 mg/100 g d.w. (1 mmol/L) to 57.35 mg/100 g d.w. (10 mmol/L). The maximum concentrations of cryptochlorogenic acid and syringic acid were similar, at 19.25 and 22.59 mg/100 g d.w., respectively. The maximum levels of vanillic, protocatechuic, and caffeic acids fluctuated between 5.23 and 13.34 mg/100 g d.w.

**Figure 5:**
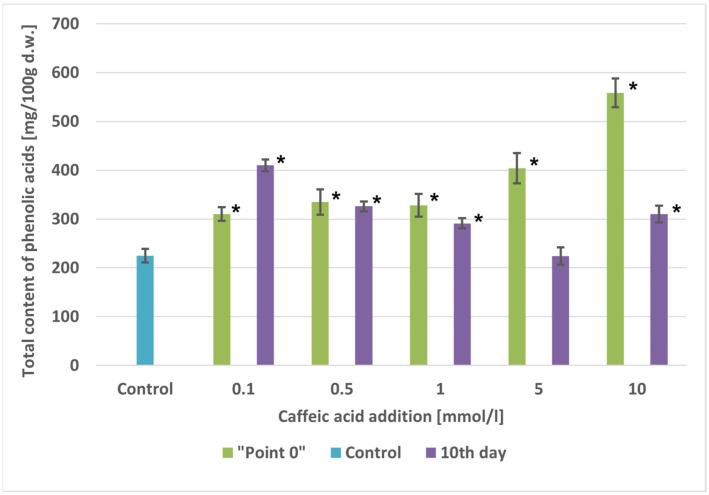
Comparison of total phenolic acids content [mg/100 g d.w.] in extracts of *A. × prunifolia* in vitro cultures supplemented with caffeic acid. * *p* < 0.05.

The maximum total contents of phenolic acids were 1.38-fold to 2.49-fold higher than in the control cultures, ranging from 310.28 mg/100 g d.w. (0.1 mmol/L) to 558.48 mg/100 g d.w. (10 mmol/L), depending on caffeic acid concentration (Figure 5).

**Table 8:** Content of phenolic acids [mg/100 g d.w.] ± SD in extracts of *A. × prunifolia* in vitro cultures supplemented with caffeic acid at “point 0”. * *p* < 0.05.

Estimated Compounds	Control	Caffeic Acid Concentration [mmol/L] Added at “Point 0”				
		0.1	0.5	1	5	10
**Neochlorogenic acid**	90.01 ± 9.97	120.24 ± 6.78 *	139.06 ± 11.61 *	139.66 ± 8.32 *	202.76 ± 14.92 *	263.54 ± 5.26 *
**Protocatechuic acid**	1.14 ± 0.08	2.94 ± 0.16 *	4.03 ± 0.19 *	4.95 ± 0.13 *	8.14 ± 0.39 *	13.34 ± 0.24 *
**Chlorogenic acid**	105.30 ± 3.06	123.45 ± 2.85 *	129.40 ± 8.97 *	132.61 ± 10.44 *	131.85 ± 11.45 *	180.22 ± 17.99 *
**Vanillic acid**	0.83 ± 0.03	1.24 ± 0.13	3.33 ± 0.18 *	2.77 ± 0.05 *	4.49 ± 0.11 *	5.23 ± 0.23 *
**Caffeic acid**	2.58 ± 0.08	7.20 ± 0.49 *	9.68 ± 0.97 *	2.09 ± 0.09	6.01 ± 0.68 *	5.61 ± 0.32 *
**Syringic acid**	11.57 ± 0.39	15.98 ± 1.09 *	15.60 ± 1.21 *	6.36 ± 0.55 *	3.80 ± 0.32 *	22.59 ± 1.64 *
**Rosmarinic acid**	6.29 ± 0.67	25.56 ± 2.11 *	21.50 ± 2.47 *	20.53 ± 2.09 *	34.49 ± 2.46 *	57.35 ± 2.95 *
**Cryptochlorogenic acid**	7.01 ± 0.19	13.67 ± 0.66 *	12.36 ± 0.33 *	19.25 ± 1.77 *	12.32 ± 0.79 *	10.61 ± 1.05 *

#### 2.6.2. Tenth Day after Culture Initiation

The quantities of chlorogenic acid and neochlorogenic acid increased 2.31-fold and 1.39-fold, respectively, depending on caffeic acid concentration, ranging from 76.87 mg/100 g d.w. (5 mmol/L) to 177.30 mg/100 g d.w. (0.1 mmol/L) for chlorogenic acid and from 103.48 mg/100 g d.w. (5 mmol/L) to 143.71 mg/100 g d.w. (0.1 mmol/L) for neochlorogenic acid (Table 9).

Rosmarinic acid contents changed within a narrow range, from 19.12 mg/100 g d.w. (0.5 mmol/L) to 25.55 mg/100 g d.w. (10 mmol/L). On the other hand, cryptochlorogenic acid contents varied more significantly, ranging from 5.62 mg/100 g d.w. (10 mmol/L) to 18.54 mg/100 g d.w. (1 mmol/L).

Among simple phenolic acids, syringic acid content varied considerably, from 2.40 mg/100 g d.w. (1 mmol/L) to 30.74 mg/100 g d.w. (0.1 mmol/L). The maximum amounts of the remaining three simple phenolic acids did not exceed 12 mg/100 g d.w.

The total contents of phenolic acids increased compared to the control cultures, rising 1.30-fold to 1.82-fold and varying from 291.08 mg/100 g d.w. (1 mmol/L) to 409.82 mg/100 g d.w. (0.1 mmol/L). However, the cultures fed with caffeic acid at 5 mmol/L were an exception, as the phenolic acid content in extracts from these cultures was identical to that of the control cultures (Figure 5).

**Table 9:** Content of phenolic acids [mg/100 g d.w.] ± SD in extracts of *A. × prunifolia* in vitro cultures supplemented with caffeic acid on the 10th day of the growth cycle. * *p* < 0.05.

Estimated Compounds	Control	Caffeic Acid Concentration [mmol/L] Added at 10th Day				
		0.1	0.5	1	5	10
**Neochlorogenic acid**	90.01 ± 9.97	143.71 ± 2.42 *	116.47 ± 2.35 *	127.86 ± 4.85 *	103.48 ± 8.96 *	120.91 ± 4.19 *
**Protocatechuic acid**	1.14 ± 0.08	4.10 ± 0.20 *	3.67 ± 0.20 *	3.04 ± 0.18 *	3.87 ± 0.10 *	4.60 ± 0.14 *
**Chlorogenic acid**	105.30 ± 3.06	177.30 ± 3.02 *	135.95 ± 3.18 *	115.54 ± 1.52 *	76.87 ± 4.97 *	133.72 ± 8.06 *
**Vanillic acid**	0.83 ± 0.03	4.26 ± 0.43 *	4.37 ± 0.28 *	2.00 ± 0.09 *	3.64 ± 0.28 *	1.36 ± 0.16
**Caffeic acid**	2.58 ± 0.08	11.82 ± 1.03 *	8.99 ± 0.16 *	2.57 ± 0.28	2.74 ± 0.25	4.67 ± 0.46 *
**Syringic acid**	11.57 ± 0.39	30.74 ± 2.03 *	23.62 ± 1.34 *	2.40 ± 0.17 *	2.97 ± 0.33 *	13.59 ± 1.30 *
**Rosmarinic acid**	6.29 ± 0.67	22.65 ± 1.63 *	19.12 ± 2.22 *	19.13 ± 1.19 *	19.60 ± 1.66 *	25.55 ± 2.38 *
**Cryptochlorogenic acid**	7.01 ± 0.19	15.24 ± 1.48 *	13.84 ± 0.29 *	18.54 ± 1.94 *	11.22 ± 1.33 *	5.62 ± 0.35

## 3. Discussion

In the present study, experimental cultures of *A. × prunifolia* fed with lower concentrations of the tested precursors showed substantial biomass increments, surpassing those in the control cultures. However, significant growth inhibition was documented at the highest concentrations of phenylalanine and caffeic acid (10 mmol/L), as well as at high concentrations (5 and 10 mmol/L) of cinnamic acid and benzoic acid. Feeding high doses of these acidic precursors to the culture media distorted the optimal pH of the media (pH 5.6–5.8), leading to marked inhibition of biomass growth (Table 1). Similar results were obtained in our previous studies on black and red aronias [23]

In the currently maintained microshoot cultures of *A. × prunifolia*, the presence of the same compounds was confirmed in both control and precursor-fed cultures. These included four depsides (chlorogenic acid, neochlorogenic acid, rosmarinic acid, and cryptochlorogenic acid) and four simple phenolic acids (protocatechuic acid, vanillic acid, caffeic acid, and syringic acid). However, precursor feeding significantly influenced the quantitative contents of these compounds. In all biomass extracts, the main compounds were chlorogenic and neochlorogenic acids (Table 2, Table 3, Table 4, Table 5, Table 6, Table 7, Table 8 and Table 9).

In stationary agar microshoot cultures of *A. × prunifolia* previously examined by our team, the quantitatively dominant compounds included rosmarinic, neochlorogenic, and chlorogenic acids [18]. On the other hand, in agitated cultures studied earlier, chlorogenic and rosmarinic acids prevailed. Notably, significant amounts of 3,4-dihydroxyphenylacetic acid were also confirmed, but the contents of neochlorogenic acid were considerably lower [17].

In the current study, extracts from agitated cultures of *A*. *× prunifolia* contained high levels of chlorogenic and neochlorogenic acids but a low level of rosmarinic acid. Additionally, the presence of 3,4-dihydroxyphenylacetic acid was not confirmed. The metabolism of *A. × prunifolia* cells was thus directed toward the production of chlorogenic and neochlorogenic acids.

The significance of the type of in vitro culture for the quality and quantitative content of different compounds has been repeatedly documented in plant biotechnology studies [21].

Differences in the metabolite profiles produced in vitro were evidenced in our earlier studies on in vitro cultures of two species of the Scutellaria genus: *S. baicalensis* and *S. lateriflora.* It was demonstrated that agar and agitated cultures maintained on variants of MS medium differed in their composition of flavonoids and phenolic acids. In contrast, on Linsmaier and Skoog (LS) [28] medium variants, the composition of compounds in agar and agitated cultures was identical [29].

Our biotechnological studies on liquid stationary and agitated cultures of *R. graveolens* and *R. graveolens* ssp. *divaricata* cultured in LS medium variants revealed an identical composition of metabolites in both types of in vitro cultures (linear furano-coumarins and umbeliferone). However, the quantities of most tested compounds were significantly higher in liquid stationary cultures compared to agitated cultures of both plants [30].

Research on dibenzocyclooctadiene lignan accumulation in agar and agitated microshoot cultures of *Schisandra chinensis* and *S. chinensis cv.* Sadova indicated a higher content of these compounds in agar cultures for both plants compared to agitated cultures [31].

Apart from the type of cultures, plant growth regulators, and light regime, many other physical factors of in vitro cultures, such as temperature, humidity, aeration, and pH of the medium, influence the biogenetic potential of cells and the production of secondary metabolites [21]. The age of the in vitro culture also plays an important role. It can be assumed that these factors influenced the changes in the metabolic pathways of purple aronia cells in the currently studied agitated cultures compared to our earlier results with agitated cultures of this plant [17].

The aim of the present study was to enhance the production of phenolic acids, including depsides in *A. × prunifolia* in vitro cultures by using a feeding strategy with their biogenetic precursors: cinnamic acid, benzoic acid, phenylalanine, and caffeic acid in the culture media.

Phenolic acids found in the plant kingdom are mostly derivatives of cinnamic acid and benzoic acid. These parent compounds are direct biosynthetic precursors of numerous phenolic acids. On the other hand, phenylalanine is a compound that appears at earlier stages of the phenolic acid biosynthetic pathway. It is also a key intermediate in the biogenesis of various other groups of phenylpropanoid metabolites, such as flavonoids, anthocyanins, catechins, lignans, phenylpropanoid glycosides, and coumarins.

These three precursors, especially phenylalanine, have been successfully used many times to stimulate phenylpropanoid production in plant in vitro cultures.

Caffeic acid molecules are structural components of depsides, and this group of compounds predominates in extracts of *A. × prunifolia* biomass. Therefore, both theoretically and practically, caffeic acid can be used as an exogenous compound to increase the production of these compounds. Our earlier experiments with black and red aronias documented that caffeic acid was a very effective precursor of depsides, especially in red aronia in vitro cultures, resulting in a maximum 5.0-fold and 5.7-fold increase in total phenolic acid content compared to black aronia cultures, which showed a 2.2-fold and 2.9-fold increase, respectively, at the 0th and 10th day of culture growth [23].

The experiments in the present study demonstrated the greatest increase in the total content of the tested compounds after feeding cinnamic acid at a concentration of 5 mmol/L, both at point “0” and on the 10th day of the culture growth cycle. This resulted in a 2.68-fold and 2.54-fold increase compared with the control cultures (Table 4, Table 5, and Table 10). The total contents obtained, amounting to ca. 603 and 571 mg/100 g d.w., were highly satisfactory and indicated significant potential for application.

Experiments on *Salvia splendens* plants also revealed that exogenous cinnamic acid efficiently stimulated the total production of phenolic acids [32]. Further, in *Larrea divaricata* cell cultures, feeding cinnamic acid on the first day of culture at a concentration of 0.5 µmol increased the production of certain phenolic acids, particularly p-coumaric acid and ferulic acid, to 225 and 50 µg/g d.w., respectively [33].

In our earlier studies on black and red aronia agitated cultures, exogenous cinnamic acid was used to boost the total production of phenolic acids. In both species, feeding this precursor at 5 mmol/L resulted in almost identical increases in their contents (3.41-fold and 3.42-fold, respectively, on the 10th day of the culture growth cycle), reaching 989.79 and 661.77 mg/100 g d.w. Feeding cinnamic acid at point “0” resulted in a smaller increase (1.90-fold and 2.10-fold) [23].

In the case of exogenous benzoic acid addition to *A. × prunifolia* cultures, a smaller maximal increase in the total phenolic acid content was observed compared with cinnamic acid: a 1.72-fold and 1.49-fold increase (0.1 mmol/L at point “0” and 0.5 mmol/L on the 10th day, yielding 387.49 and 334.31 mg/100 g d.w., respectively). In *Nicotiana tabacum cv. Samsun NN* suspension cultures, benzoic acid (100 µmol) proved to be an efficient precursor of salicylic acid (o-hydroxybenzoic acid), yielding a maximum of 16 µg/g f.w. [34].

Correspondingly, our experiments with black and red aronia agitated cultures also showed a much weaker ability of benzoic acid to elevate the total production of phenolic acids, including depsides, compared with exogenous cinnamic acid: a maximum 1.73-fold increase (1 mmol/L, 10th day) and a maximum 1.81-fold increase (1 mmol/L, at point “0” and the 10th day), respectively [23].

In the current *A. × prunifolia* cultures fed with phenylalanine, the stimulatory effects on the total production of the tested compounds were satisfactory, with a 2.06-fold and 1.98-fold increase—reaching 463.77 mg/100 g d.w. (5 mmol/L, point “0”) and 445.99 mg/100 g d.w. (1 mmol/L, 10th day), respectively (Table 2, Table 3, and Table 10).

In our earlier experiments on phenylalanine-fed black aronia cultures, a similar 2.0-fold maximum increase in the total production of phenolic acids was documented (0.1 mmol/L, 10th day). An even greater 2.6-fold and 2.8-fold increase in total phenolic acid content was observed in red aronia cultures at point “0” and on the 10th day of culture, respectively (1 mmol/L) [23].

In more recent investigations by our team focused on *Gingko biloba* suspension cultures, we demonstrated the beneficial effect of exogenous phenylalanine (tested at concentrations of 100, 150, and 200 mg/150 mL of culture media) on phenolic acid production. In the cultures maintained for 3 weeks, the greatest phenolic acid content (73.76 mg/100 g d.w.) was obtained 4 days after feeding with phenylalanine (200 mg/150 mL). Protocatechuic acid and p-hydroxybenzoic acid were the main accumulated compounds [25].

On the other hand, exogenous phenylalanine (1.25 g/L) primarily stimulated the production of p-coumaric acid and ferulic acid in agitated microshoot cultures of *Ruta graveolens* maintained in our laboratory (64.3 and 35.6 mg/100 g d.w., respectively). The best effect was observed in 4-week-old cultures on the second day after precursor addition, with the total content of phenolic acids reaching a maximum of 109.00 mg/100 g d.w. Feeding with phenylalanine also increased catechin production (maximum 66.00 mg/100 g d.w.) on the third day after treatment [26].

Exogenous phenylalanine (1 g/L) was also tested by our team as a biogenetic precursor of phenolic acids in agitated microshoot cultures of three *Hypericum perforatum* cultivars—Elixir, Helos, and Topas. The total phenolic acid content reached its maximum after 7 days, with Elixir cv. at 771 mg/100 g d.w., Helos cv. at 662 mg/100 g d.w., and Topas cv. at 613 mg/100 g d.w., respectively. The maximal phenolic acid content increased 1.73-fold, 1.31-fold, and 1.61-fold, respectively. The qualitative composition of phenolic acids in the biomass extracts, after feeding the culture media with the precursor, was richer. Additionally, the presence of p-hydroxybenzoic acid, p-coumaric acid, and vanillic acid (the latter found only in selected extracts) was confirmed. The supplementation also enhanced the total content of flavonoids and catechins by 2.33-fold and 1.33-fold, respectively [27].

Similarly, phenylalanine feeding (at 1.0–2.5 g/L) in agitated microshoot cultures of *Scutellaria lateriflora* stimulated the production of specific Scutellaria flavonoids and verbascoside. The addition of phenylalanine (at a concentration of 1.5 g/L) most efficiently boosted the production of these compounds (maximum 3765 and 475 mg/100 g d.w. after 7 days, respectively), outperforming the addition of different concentrations of tyrosine (1.0–2.5 g/L) or elicitation with methyl jasmonate (10, 50, and 100 µmol), and proving more effective than concomitant feeding with the above-mentioned amino acids and elicitation [27].

Studies by other research teams have analogously documented the favorable influence of exogenous phenylalanine on the production of different groups of phenylpropanoids. In suspension cultures of *Linum flavum* maintained in medium containing NAA, a 3-fold to 5-fold increase in the content of 5-methoxypodophyllotoxin was observed after 8–11 days. On NAA-free medium, the increase was spectacular, with an approximately 40-fold to 50-fold higher content of this lignan estimated (0.161% d.w.) [35].

Comparably, in *Abutilon indicum* callus cultures maintained with exogenous phenylalanine (50–100 mg/100 mL), quercetin content rose by a maximum of 3-fold (75 mg/100 mL), reaching 0.47 mg/g d.w. (control 0.14 mg/g d.w.) in 4-week-old cultures [36]. In contrast, in liquid cell cultures of *Citrullus colocynthis*, the highest 2.3-fold increase in total quercetin content (free and bound) was confirmed at lower concentrations of exogenous phenylalanine (50 mg/100 mL, 7.25 mg/g d.w., control 3.05 mg/g d.w.). The lowest amounts of this flavonoid were confirmed after supplementation of the culture medium with the highest concentration of precursor—4.70 and 4.08 mg/g d.w. at 70 and 100 mg/100 mL, respectively [37].

In *Cistanche deserticola* suspension cultures, supplementation with 0.2 mmol/L of phenylalanine (on the eighth day of the growth cycle) increased the production of phenylpropanoid glycosides by up to 175% compared to the control culture, reaching 18.6 g%. The experiments also documented that phenylalanine was the best precursor among the parallel-tested tyrosine and phenylacetic acid. Phenylalanine feeding experiments were also performed in large-scale cultures of *C. deserticola*—in 2 l bubble column bioreactors, a 160% increase in phenylethanoid glycosides was achieved after 20 days, amounting to about 14.2% [38].

Exogenous phenylalanine (0.1 mmol) also stimulated the production of phenolic acids in suspension cultures of *Vitis vinifera* cv. Muscat de Frontignan, where a 1.5-fold increase in production was confirmed after 4 days. A similarly satisfactory effect was observed with shikimic acid feeding as a precursor (0.1 mmol) 2 days after supplementation [39].

The addition of exogenous caffeic acid (10 mmol/L, at “point 0”) to *A. × prunifolia* cultures resulted in a significant 2.49-fold increase in total phenolic acid production, reaching 558.48 mg/100 g d.w. (Table 8 and Table 10).

In our earlier experiments on red aronia cultures, caffeic acid at a concentration of 5 mmol/L caused a substantial 5.67-fold increase in the production of phenolic acids, including depsides. In contrast, in black aronia cultures, caffeic acid at the same concentration (5 mmol/L) induced a 2.95-fold increase in the total content of phenolic acids, particularly depsides [23].

In the current *A. × prunifolia* cultures fed with two exogenous biogenetic precursors—benzoic acid and caffeic acid—we observed a generally stronger stimulatory effect on total phenolic acid production when these precursors were added to MS medium at point “0.” In contrast, when phenylalanine and cinnamic acid were used as precursors, the amounts of phenolic acids were generally higher when these compounds were added on the 10th day of culture (Table 2, Table 3, Table 4, Table 5, Table 6, Table 7, Table 8, Table 9 and Table 10). These effects differ from those observed in previously tested black and red aronia cultures. In black aronia cultures, the 10th day of culture was generally the better time for supplementation with all precursors. On the other hand, in red aronia cultures, for certain concentrations of phenylalanine and cinnamic acid (0.1 and 0.5 mmol/L) and caffeic acid (0.1 and 4.0 mmol/L), the better time for media supplementation was point “0” [23].

This short review of feeding experiment results concludes that identifying optimal feeding conditions—such as the type of precursor, its concentration, timing of supplementation, and timing of biomass collection—is essential for obtaining applicable results.

Summarizing the current results, we propose that the optimal conditions for stimulating the total production of phenolic acids in *A. × prunifolia* cultures fed with exogenous precursors are achieved by adding cinnamic acid at 5 mmol/L at both point “0” and on the 10th day of culture (603.03 and 570.57 mg/100 g d.w., respectively), and by adding caffeic acid at 10 mmol/L on the 10th day of culture growth (558.48 mg/100 g d.w.).

Similarly, in in vitro cultures of black and red aronia, cinnamic acid and caffeic acid were also the most effective among the four tested precursors for stimulating the production of phenolic acids [23].

Among the tested compounds, two depsides—chlorogenic acid and neochlorogenic acid—were predominant in *A. × prunifolia* biomass extracts. These compounds show invaluable biological activities, including antioxidant properties, as well as anti-inflammatory, anticancer, neuroprotective, cardioprotective, hepatoprotective, and antimicrobial actions. They also influence lipid and glucose metabolism [40,41,42,43]. The highest contents of these depsides achieved after feeding the four tested precursors to the media were 278.96, 388.29, 167.66, and 180.00 mg/100 g d.w. (control cultures: 105.30 mg/100 g d.w.) for chlorogenic acid, and 146.65, 146.53, 148.49, and 263.54 mg/100 g d.w. (control cultures: 90.01 mg/100 g d.w.) for neochlorogenic acid, respectively. We propose that feeding cinnamic acid at a concentration of 5 mmol/L at point “0” is optimal for maximizing chlorogenic acid production while feeding caffeic acid at 10 mmol/L at point “0” is optimal for stimulating neochlorogenic acid production.

A more practical and universal approach for achieving the highest production of these two depsides would be to maintain agitated cultures of *A. × prunifolia* enriched with cinnamic acid at 5 mmol/L at point “0”.

The precursors tested in the present experiments are readily available and inexpensive chemical compounds. Therefore, they can be tested in scaled-up cultures, such as in bioreactor systems (e.g., commercially available TIS (Temporary Immersion Systems) bioreactors like RITA^®^ or PlantForm dedicated to microshoot cultures). Initial experiments conducted by our team using PlantForm bioreactors with *A*. × *prunifolia* cultures have shown promising results. However, it is necessary to verify the repeatability of these findings.

## 4. Materials and Methods

### 4.1. Establishment of Agar Basal Microshoot Cultures

The basal microshoot cultures of *A.* × *prunifolia* (Marsh.) Rhed. were established from leaf buds of plants sourced from the Arboretum in Rogów (Poland), a unit of Warsaw University of Life Sciences, Forest Experimental Station in Rogów (see Szopa et al., 2018 [18] for details).

### 4.2. Agitated Experimental Microshoot Cultures

The agitated experimental microshoot cultures of *A.* × *prunifolia* were derived from agar basal cultures. These cultures were maintained in 300 mL Erlenmeyer flasks containing MS [20] medium enriched with 1 mg/L 6-benzylaminopurine (BAP) and 1 mg/L 1-naphthaleneacetic acid (NAA). Each flask contained 2 g of fresh microshoots (inoculum) and 100 mL of MS medium.

The cultures were grown on a rotary shaker (Altel Company, Poland) at 140 rpm for a 20-day growth cycle.

At the start (point “0”) and after 10 days of growth, four different phenolic acids and depside precursors (phenylalanine, cinnamic acid, benzoic acid, and caffeic acid) were added to the culture media at five different concentrations: 0.1, 0.5, 1.0, 5.0, and 10.0 mmol/L.

Control cultures (without the addition of precursors) were maintained independently.

The different concentrations of precursors were achieved by adding their respective 0.1 mmol/mL stock solutions and sterile redistilled water to flasks with MS medium (for details, see [23]).

### 4.3. Phytochemical Analysis

After 20 days of growth, the microshoots were separated from the medium, dried in a lyophilizer (Labconco, Kansas City, USA), and powdered. A 0.5 g sample was extracted with analytical-grade methanol using a reflux condenser (twice, with 50 mL each, for 2 h).

The extracts were evaporated to a constant mass, and the residues were dissolved in 2 mL of methanol (HPLC grade, Merck, Darmstadt, Germany).

Experimental media (40 mL) were collected, dried in a lyophilizer, and then dissolved in 5 mL of methanol.

The estimation of phenolic acids and depsides was performed by HPLC analysis according to Ellnain-Wojtszek and Zgórka [44] using a Merck-Hitachi apparatus with a pump L2130 and DAD detector L2455. The analysis was conducted with an RP−18 e column (Merck, 250 × 4 mm, 5 µm) at 25 °C. The mobile phases used were methanol (A) and methanol: 0.5% acetic acid (1:4 *v*/*v*) (B). Gradient elution was used as follows: 0–20 min, 100% B; 20–35 min, 80% B; 35–45 min, 70% B; 45–55 min, 60% B; 55–60 min, 50% B; 60–65 min, 25% B; 65–75 min, 0% B; and 75–90 min, 100% B. The flow rate was 1 mL/min, the injection volume was 10 µL, and the detector was set to 254 nm.

Quantitative analysis was based on comparing the peak areas with standard calibration curves. The following standards of phenolic acids and depsides were used:

- Cinnamic acid derivatives: caffeic, caftaric, m-coumaric, o-coumaric, p-coumaric, ferulic, gentisic, hydrocaffeic, isoferulic, synapic, and vanillic acids;
- Benzoic acid derivatives: gallic, p-hydroxybenzoic, protocatechuic, salicylic, and syringic acids;
- Phenylacetic acid derivatives: 3,4-dihydroxyphenylacetic acid;
- Depsides: chlorogenic, cryptochlorogenic, isochlorogenic, neochlorogenic, rosmarinic, and ellagic acids.

Additionally, standards of the parent compounds of phenolic acid subgroups (cinnamic, benzoic, and phenylacetic acids) and phenylalanine were used. All standards were purchased from Sigma-Aldrich, St. Louis, MO, USA.

### 4.4. Statistical Analysis

Statistical analysis was performed with the STATISTICA 12.0 software package (StatSoft. Inc., Tulsa, OK, USA). All data are expressed as mean values ± standard deviation (±SD). The Student’s *t*-test was used to compare the results with the control samples. The statistical significance was set at *p* < 0.05. The results are presented as the mean values from three independent experiments in three repetitions (*n* = 5). Error bars indicate standard deviations. Significant difference is marked with an asterisk “*” (*p* < 0.05).

## 5. Conclusions

The results of the extensive research with agitated microshoot cultures of *A. × prunifolia* documented that feeding the culture media with two of the four tested biogenetic precursors—cinnamic acid and caffeic acid—resulted in high production of two depsides: chlorogenic and neochlorogenic acids. These findings are an important step toward proposing *A. × prunifolia* microshoot extracts as a rich source of these bioactive compounds, which have high antioxidant activity, for use in phytopharmaceuticals, health foods, and phytocosmetics. However, scaling up the in vitro cultures to bioreactors is necessary for practical application.

## Figures and Tables

**Table 10:** Effect of feeding strategy in *A* × *prunifolia* agitated microshoot cultures—the increase in the total contents of phenolic acids vs. control cultures.

Precursor	Increase in Total Content of Phenolic Acids vs. Control									
	0.1 mmol/L		0.5 mmol/L		1.0 mmol/L		5.0 mmol/L		10.0 mmol/L	
	Point “0”	10th Day	Point “0”	10th Day	Point “0”	10th Day	Point “0”	10th Day	Point “0”	10th Day
**Phenylalanine**	1.22	1.32	1.02	1.48	1.51	1.98	2.06	1.82	↓1.38	1.47
**Cinnamic acid**	1.24	1.24	1.38	1.95	1.92	1.86	2.68	2.54	↓2.22	1.67
**Benzoic acid**	1.72	1.10	1.03	1.49	1.44	1.04	↓1.89	↓2.79	↓1.22	↓2.09
**Caffeic acid**	1.38	1.82	1.49	1.45	1.46	1.30	1.80	↓1.0	2.49	1.38

## Data Availability

Data are contained within the article.

## References

[1] Natella F., Nardini M., Di Felice M., Scaccini C. (1999). Benzoic and Cinnamic Acid Derivatives as Antioxidants: Structure-Activity Relation. J. Agric. Food Chem..

[2] Heleno S.A., Martins A., Queiroz M.J.R.P., Ferreira I.C.F.R. (2015). Bioactivity of Phenolic Acids: Metabolites versus Parent Compounds: A Review. Food Chem..

[3] Khan M.K., Paniwnyk L., Hassan S., Li Y.C.F. (2019). Polyphenols as Natural Antioxidants: Sources, Extraction and Applications in Food, Cosmetics and Drugs. Plant Based “Green Chemistry 2.0”: Moving from Evolutionary to Revolutionary.

[4] Mehla N., Kothari Chhajer A., Kumar K., Dahiya S., Mohindroo V., Ekiert H.M., Ramawat K.G., Arora J. (2021). Applications of Antioxidants: A Review. Plant Antioxidants and Health. Reference Series in Phytochemistry.

[5] Cai Y., Luo Q., Sun M., Corke H. (2004). Antioxidant Activity and Phenolic Compounds of 112 Traditional Chinese Medicinal Plants Associated with Anticancer. Life Sci..

[6] Piazzon A., Vrhovsek U., Masuero D., Mattivi F., Mandoj F., Nardini M. (2012). Antioxidant Activity of Phenolic Acids and Their Metabolites: Synthesis and Antioxidant Properties of the Sulfate Derivatives of Ferulic and Caffeic Acids and of the Acyl Glucuronide of Ferulic Acid. J. Agric. Food Chem..

[7] Robbins R.J. (2003). Phenolic Acids in Foods: An Overview of Analytical Methodology. J. Agric. Food Chem..

[8] Khadem S., Marles R.J. (2010). Monocyclic Phenolic Acids; Hydroxy- and Polyhydroxybenzoic Acids: Occurrence and Recent Bioactivity Studies. Molecules.

[9] Matkowski A. (2008). Plant in Vitro Culture for the Production of Antioxidants—A Review. Biotechnol. Adv..

[10] Ekiert H.M., Kubica P., Kwiecień I., Jafernik K., Klimek-Szczykutowicz M., Szopa A., Ekiert H.M., Ramawat K.G., Arora J. (2022). Cultures of Medicinal Plants In Vitro as a Potential Rich Source of Antioxidants. Plant Antioxidants and Health. Reference Series in Phytochemistry.

[11] Georgiev V., Slavov A., Vasileva I., Pavlov A. (2018). Plant Cell Culture as Emerging Technology for Production of Active Cosmetic Ingredients. Life Sci..

[12] Krasteva G., Georgiev V., Pavlov A. (2021). Recent Applications of Plant Cell Culture Technology in Cosmetics and Foods. Eng. Life Sci..

[13] Eibl R., Meier P., Stutz I., Schildberger D., Hühn T., Eibl D. (2018). Plant Cell Culture Technology in the Cosmetics and Food Industries: Current State and Future Trends. Appl. Microbiol. Biotechnol..

[14] Celka Z., Szkudlarz P. (2010). Spontaneous Occurrence and Dispersion of *Aronia x Prunifolia* (Marshall) Rehder (Rosaceae) in Poland on the Example of the “Bagna” Bog Complex near Chlebowo (Western Poland). Acta Soc. Bot. Pol..

[15] Szopa A., Kokotkiewicz A., Kubica P., Banaszczak P., Wojtanowska-Krośniak A., Krośniak M., Marzec-Wróblewska U., Badura A., Zagrodzki P., Bucinski A. (2017). Comparative Analysis of Different Groups of Phenolic Compounds in Fruit and Leaf Extracts of *Aronia* sp.: *A. Melanocarpa*, *A. Arbutifolia* and *A. x Prunifolia*, and Their Antioxidant Activities. Eur. Food Res. Technol..

[16] Taheri R., Connolly B.A., Brand M.H., Bolling B.W. (2013). Underutilized Chokeberry (*Aronia melanocarpa, Aronia arbutifolia, Aronia prunifolia*) Accessions Are Rich Sources of Anthocyanins, Flavonoids, Hydroxycinnamic Acids, and Proanthocyanidins. J. Agric. Food Chem..

[17] Szopa A., Kubica P., Ekiert H. (2018). Agitated Shoot Cultures of *Aronia arbutifolia* and *Aronia × Prunifolia*: Biotechnological Studies on the Accumulation of Phenolic Compounds and Biotransformation Capability. Plant Cell Tissue Organ. Cult..

[18] Szopa A., Kubica P., Snoch A., Ekiert H. (2018). High Production of Bioactive Depsides in Shoot and Callus Cultures of *Aronia arbutifolia* and *Aronia × Prunifolia*. Acta Physiol. Plant.

[19] Szopa A., Starzec A., Ekiert H. (2018). The Importance of Monochromatic Lights in the Production of Phenolic Acids and Flavonoids in Shoot Cultures of *Aronia melanocarpa*, *Aronia arbutifolia* and *Aronia × Prunifolia*. J. Photochem. Photobiol. B.

[20] Murashige T., Skoog F. (1962). A Revised Medium for Rapid Growth and Bioassays with Tobacco Tissue Cultures. Physiol. Plant.

[21] Ramawat K.G., Mathur M., Ramawat K.G., Merillon J.M. (2007). Factors Affecting the Production of Secondary Metabolites. Biotechnology: Secondary Metabolites, Plants and Microbes.

[22] Wysokińska H., Chmiel A. (1997). Transformed Root Cultures for Biotechnology. Acta Biotechnol..

[23] Szopa A., Kubica P., Komsta Ł., Walkowicz-Bożek A., Ekiert H. (2020). The Effect of Feeding Culture Media with Biogenetic Precursors on High Production of Depsides in Agitated Shoot Cultures of Black and Red Aronias. Plant Cell Tissue Organ. Cult..

[24] Kwiecień I., Miceli N., Kędzia E., Cavò E., Taviano M.F., Beerhues L., Ekiert H. (2023). Different Types of *Hypericum perforatum cvs* (Elixir, Helos, Topas) In Vitro Cultures: A Rich Source of Bioactive Metabolites and Biological Activities of Biomass Extracts. Molecules.

[25] Szewczyk A., Kwiecień I., Grabowski M., Rajek K., Cavò E., Taviano M.F., Miceli N. (2021). Phenylalanine Increases the Production of Antioxidant Phenolic Acids in *Ginkgo biloba* Cell Cultures. Molecules.

[26] Szewczyk A., Paździora W., Ekiert H. (2023). The Influence of Exogenous Phenylalanine on the Accumulation of Secondary Metabolites in Agitated Shoot Cultures of *Ruta graveolens* L.. Molecules.

[27] Kwiecień I., Miceli N., D’arrigo M., Marino A., Ekiert H. (2022). Antioxidant Potential and Enhancement of Bioactive Metabolite Production in In Vitro Cultures of *Scutellaria lateriflora* L. by Biotechnological Methods. Molecules.

[28] Linsmaier E.M., Skoog F. (1965). Organic Growth Factor Requirements of Tobacco Tissue Cultures. Physiol. Plant.

[29] Kawka B., Kwiecień I., Ekiert H., Ramawat K., Ekiert H., Goyal S. (2019). Production of Specific Flavonoids and Verbascoside in Shoot Cultures of *Scutellaria baicalensis*. Plant Cell and Tissue Differentiation and Secondary Metabolites.

[30] Ekiert H., Czygan F.C. (2005). Accumulation of Biologically Active Furanocoumarins in Agitated Cultures of *Ruta graveolens* L. and *Ruta graveolens* ssp. *Divaricata* (Tenore) Gams. Pharmazie.

[31] Szopa A., Kokotkiewicz A., Klimek-Szczykutowicz M., Luczkiewicz M., Ekiert H., Ramawat K., Ekiert H., Goyal S. (2021). Different Types of in Vitro Cultures of *Schisandra chinensis* and Its Cultivar (*S. chinensis cv. Sadova*): A Rich Potential Source of Specific Lignans and Phenolic Compounds. Plant Cell and Tissue Differentiation and Secondary Metabolites.

[32] McCalla D.R., Neish A.C. (1959). Metabolism of Phenylpropanoid Compounds in Salvia: II.Biosynthesis of Phenolic Cinnamic Acids. Can. J. Biochem. Physiol..

[33] Palacio L., Cantero J.J., Cusidó R., Goleniowski M. (2011). Phenolic Compound Production by *Larrea divaricata* Cav. Plant Cell Cultures and Effect of Precursor Feeding. Process Biochem..

[34] Chong J., Pierrel M.A., Atanassova R., Werck-Reichhart D., Fritig B., Saindrenan P. (2001). Free and Conjugated Benzoic Acid in Tobacco Plants and Cell Cultures. Induced Accumulation upon Elicitation of Defense Responses and Role as Salicylic Acid Precursors. Plant Physiol..

[35] van Uden W., Pras N., Vossebeld E.M., Mol J.N.M., Malingré T.M. (1990). Production of 5-Methoxypodophyllotoxin in Cell Suspension Cultures of *Linum flavum* L.. Plant Cell Tissue Organ. Cult..

[36] Sajjalaguddam R.R., Paladugu A. (2015). Phenylalanine Enhances Quercetin Content in in Vitro Cultures of *Abutilon indicum* L.. J. Appl. Pharm. Sci..

[37] Chand Meena M., Kesh Meena R., Patni V. (2014). Effect of Elicitor on Quercetin Production in Cell Cultures of *Citrullus colocynthis* (Linn.) Schrad. Pharma Innov.-J..

[38] Ouyang J., Wang X.D., Zhao B., Wang Y.C. (2005). Enhanced Production of Phenylethanoid Glycosides by Precursor Feeding to Cell Culture of *Cistanche deserticola*. Process Biochem..

[39] Riedel H., Akumo N., Min N., Saw M.T., Kütük O., Neubauer P., Smetanska I. (2012). Elicitation and Precursor Feeding Influence Phenolic Acids Composition in *Vitis vinifera* Suspension Culture. Afr. J. Biotechnol..

[40] Sato Y., Itagaki S., Kurokawa T., Ogura J., Kobayashi M., Hirano T., Sugawara M., Iseki K. (2011). In Vitro and in Vivo Antioxidant Properties of Chlorogenic Acid and Caffeic Acid. Int. J. Pharm..

[41] Meng S., Cao J., Feng Q., Peng J., Hu Y. (2013). Roles of Chlorogenic Acid on Regulating Glucose and Lipids Metabolism: A Review. Evid.-Based Complement. Altern. Med..

[42] Rosa L., Silva N., Soares N., Monteiro M., Teodoro A. (2016). Anticancer Properties of Phenolic Acids in Colon Cancer—A Review. J. Nutr. Food Sci..

[43] Naveed M., Hejazi V., Abbas M., Kamboh A.A., Khan G.J., Shumzaid M., Ahmad F., Babazadeh D., FangFang X., Modarresi-Ghazani F. (2018). Chlorogenic Acid (CGA): A Pharmacological Review and Call for Further Research. Biomed. Pharmacother..

[44] Ellnain-Wojtaszek M., Zgórka G. (1999). High-Performance Liquid Chromatography and Thin-Layer Chromatography of Phenolic Acids from *Ginkgo biloba* L. Leaves Collected within Vegetative Period. J. Liq. Chromatogr. Relat. Technol..

